# A homozygous mutation of alanyl‐transfer RNA synthetase 2 in a patient of adult‐onset leukodystrophy: A case report and literature review

**DOI:** 10.1002/brb3.1313

**Published:** 2019-05-20

**Authors:** Jian‐Yong Wang, Song‐Fang Chen, Hong‐Qiu Zhang, Meng‐Yan Wang, Jian‐Hong Zhu, Xiong Zhang

**Affiliations:** ^1^ Department of Geriatrics & Neurology The Second Affiliated Hospital and Yuying Children's Hospital, Wenzhou Medical University Wenzhou Zhejiang China; ^2^ Department of Preventive Medicine, School of Public Health Wenzhou Medical University Wenzhou Zhejiang China

**Keywords:** AARS2, alanyl‐tRNA synthetase, leukodystrophy, leukoencephalopathy, mutation

## Abstract

**Introduction:**

Leukodystrophy is a group of hereditary leukoencephalopathies predominantly affecting the white matter. Multiple genes and mutations have been reported to be associated with this disorder. Identification of pathogenic genes can facilitate diagnosis of leukodystrophy and development of therapeutic strategies.

**Methods:**

A case was presented with clinical examinations. Exome sequencing was applied to identify potential mutations. Sanger sequencing of blood DNA was applied to confirm the mutation and to examine additional members.

**Results:**

We reported a Chinese male patient of adult‐onset leukodystrophy. Genetic examinations identified a homozygous mutation, c. 452T>C (p. M151T), in alanyl‐tRNA synthetase 2 (*AARS2*) in the patient. The disease was autosomal recessive as suggested by the genotypic analyses of his family members. We also reviewed phenotypic spectra of *AARS2* mutation‐associated leukodystrophies from a total of 16 reported cases.

**Conclusions:**

Our data provide further evidence that mutations of *AARS2* are implicated in adult‐onset leukodystrophy.

## 1 BACKGROUND

Leukodystrophy is a group of hereditary leukoencephalopathies characterized by lesions in white matter (van der Knaap & Bugiani, 2017; Vanderver et al., 2015), which can be detected by magnetic resonance imaging (MRI) (Di Donato, Dotti, & Federico, 2014). This disease is manifested by pyramidal and extrapyramidal signs, mental and behavior disorders, cognitive decline (Lynch et al., 2016). The phenotypes are variable subjecting to interactions between genetic mutations and environmental exposure (Kaye & Moser, 2004). Mutations in *GFAP*, *EIF2B1*, *ARSA*, *ABCD1,* and *MLC1* have been identified in leukodystrophies (Renaud, 2012). Discovery of additional pathogenic genes may facilitate diagnosis and treatment of leukodystrophies.

In this study, we reported a Chinese patient who was diagnosed as adult‐onset leukodystrophy. We unveiled a unique homozygous mutation in alanyl‐transfer RNA (tRNA) synthetase 2 (*AARS2*) (OMIM #612035) in this patient.

## 2 CASE PRESENTATION

The patient was a 44‐year‐old Chinese man who was born healthy and developed normally until the age of 40 when he felt difficult to walk straight. The patient displayed cognitive decline and behavioral abnormality when he first sought medical advices associated with this discomfort at the age of 43 at the Second Affiliated Hospital and Yuying Children's Hospital, Wenzhou Medical University. Neurological examinations revealed bilateral cerebellar ataxia and generalized hyperreflexia. The Mini‐Mental State Examination score of the patient was 12/30. His muscle bulk, tone, strength, and sensory function were normal. No abnormality in electrocardiogram and echocardiography was found. No endocrine disorder was detected. The Laboratory parameters including plasma lactate, complete blood count, and cerebrospinal fluid protein content were normal. His parents are non‐consanguineous. His grandparents, parents, and his elder brother did not show any neurological or psychiatric problems.

**Figure 1:**
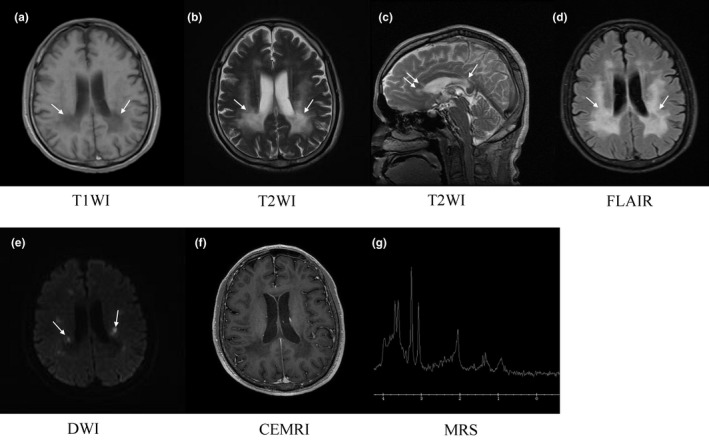
Brain magnetic resonance imaging (MRI) of the patient. (a) T1‐weighted image, showing hypointensity of periventricular white matter. (b) T2‐weighted image, showing hyperintensity in the deep and periventricular white matter. (c) Sagittal view of T2‐weighted image, showing multiple segments of high signal in the corpus callosum. (d) Fluid attenuated inversion recovery (FLAIR), showing hyperintensity in the deep and periventricular white matter. (e) Diffusion‐weighted imaging (DWI), showing sporadic restricted diffusion abnormalities in periventricular white matter. (f) Contrast enhanced MRI (CEMRI), showing no enhanced signal. (g) Magnetic resonance spectroscopy (MRS), showing high levels of choline and low levels of *N*‐acetylaspartate in the left periventricular brain tissue. Arrows indicate the lesion sites or abnormal signals

The T1‐ and T2‐weighted image of brain magnetic resonance imaging (MRI) exhibited extensive symmetric periventricular white matter abnormalities with slight ventricular system enlargement and sulci widening. The midline structure remained unchanged (Figure 1a,b). Multiple segments of high signal in the corpus callosum were detected in the sagittal view of T2‐weighted image (Figure 1c). Analyses by fluid attenuated inversion recovery (FLAIR) showed hyperintensity in the deep and periventricular white matter, indicating white matter rarefaction (Figure 1d). Diffusion‐weighted imaging (DWI) showed sporadic restricted diffusion abnormalities in periventricular white matter (Figure 1e). No enhanced signal was found in contrast enhanced MRI (CEMRI; Figure 1f). Magnetic resonance spectroscopy (MRS) showed a relatively high level of choline and low level of *N*‐acetylaspartate in the left periventricular brain tissue (Figure 1g), where the T2‐weighted hyperintensity was detected (Figure 1b).

**Figure 2:**
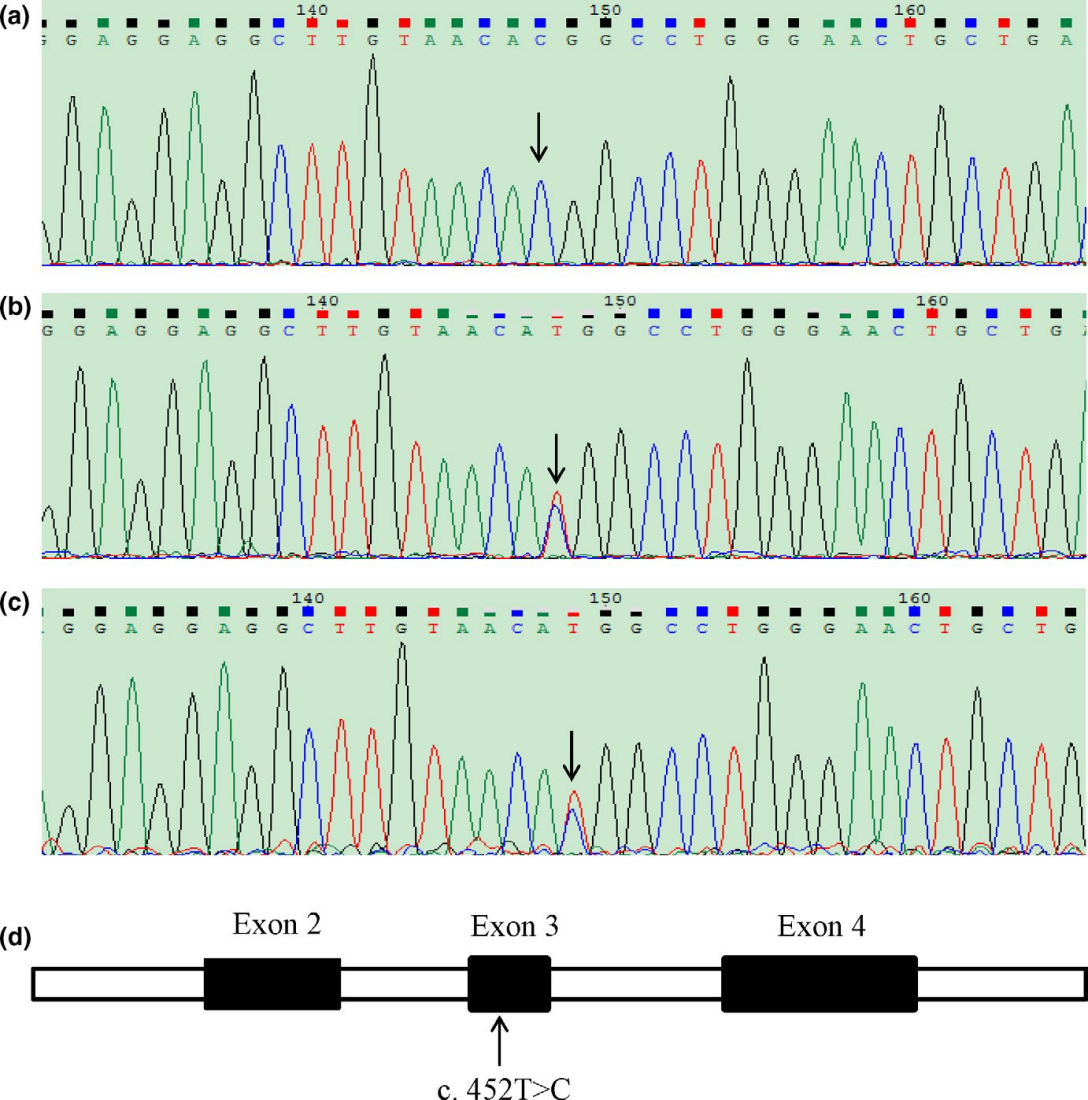
Sanger sequencing of the patient and his family members. (a) Homozygous mutation of C. 452T>C in *AARS2* in the patient. (b‐c) Heterozygous mutation of C. 452T>C in his mother (b) and brother (c). (d) Schematic location of the C. 452T>C mutation in *AARS2*

**Figure 3:**
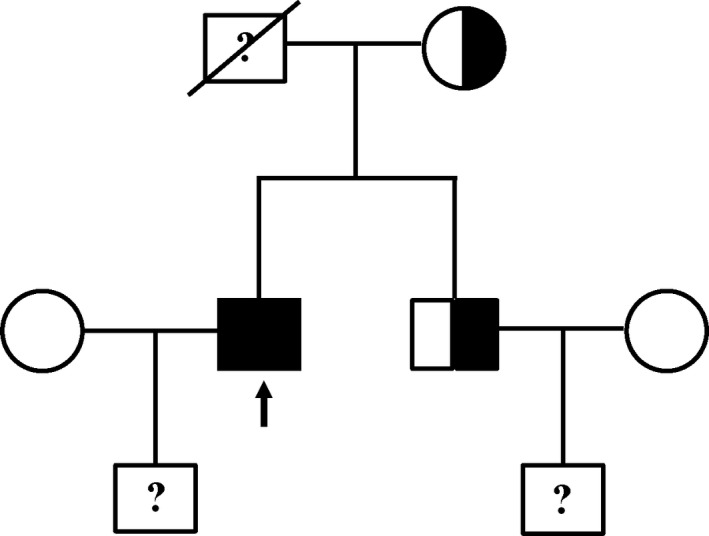
Pedigree of the family. Arrow indicates the patient; question mark indicates non‐sequence

Exome sequencing revealed that the patient carried a homozygous mutation in *AARS2*, that is, c. 452T>C (p. M151T). The mutation was confirmed by Sanger sequencing of his blood DNA (Figure 2a). The patient's father passed away at the age of 62 with no record of neurological disorders. We sequenced and analyzed the *AARS2* genotype of his mother and elder brother. The results showed that both of them were heterozygous (Figure 2b,c). Thus, this patient in principle maternally and paternally inherited the mutation to be homozygous (Figure 3).

The mutation was missense and localized in the exon 3 of *AARS2* (Figure 2d). There was no record of this mutation in dbSNP (https://www.ncbi.nlm.nih.gov/SNP/). The mutation was “probably damaging” as predicted by PolyPhen‐2 (http://genetics.bwh.harvard.edu/pph2/) and ascribed to the category of “affected protein function” based on SIFT (http://sift.jcvi.org).

Donepezil at the dose of 5 mg/day was prescribed to the patient for a duration of 1 month. Regular exercise such as walking was suggested. No significant improvement in cognitive function was observed in his follow‐up visits.

## 3 DISCUSSION

Leukoencephalopathy is a broad term that predominantly affects the white matter in association with multiple types of disorders, including infectious, inflammatory, vascular, and hereditary (Costello, Eichler, & Eichler, 2009; Vanderver et al., 2015). Leukodystrophy is generally referred to the hereditary type of leukoencephalopathy (Vanderver et al., 2015), and there is no effective treatment. In this study, we reported a unique case of adult‐onset leukodystrophy with a homozygous mutation of c. 452T>C (p. M151T) in *AARS2*.

*AARS2* encodes mitochondrial alanyl‐tRNA synthetase, which is responsible for the aminoacylation between alanine and the tRNA during translation in the mitochondria. Deficiency in aminoacyl‐tRNA synthetases is known to contribute to mitochondrial diseases in association with a wide spectrum of clinical phenotypes (Fuchs et al., 2018; Konovalova & Tyynismaa, 2013). The AARS2 protein contains an editing and an aminoacylation domain, and mutations site‐specifically change its function in the catalysis of aminoacylation (Euro et al., 2015).

*AARS2* mutations were first identified in infantile mitochondrial cardiomyopathy in 2011 (Gotz et al., 2011), and later found to cause two different phenotypic disorders: severe infantile cardiomyopathy and adult‐onset leukodystrophy (Dallabona et al., 2014). Strikingly, no cardiopathy is present in adult‐onset leukodystrophy as suggested in our patient and 15 other reported cases (Table 1) (Dallabona et al., 2014; Hamatani et al., 2016; Lee et al., 2017; Lynch et al., 2016; Szpisjak et al., 2017). To date, all of the infantile cardiomyopathy exhibit a founder mutation, c. 1774C>T (p. R592W), which is located in the editing domain and severely compromises the aminoacylation activity of AARS2 (Euro et al., 2015). In comparison, mutations in adult‐onset leukodystrophy include combinations of two missense mutations in the aminoacylation domain, a missense mutation in the aminoacylation domain with a truncating mutation, and other combinations. These mutations can be classified into three categories of activity impairment based on structural analyses, that is, loss of function, severely impaired, and moderate impaired (Euro et al., 2015). The extent of reduced aminoacylation activities may lead to differential phenotypes.

**Table 1:** Characteristics of adult‐onset leukodystrophy induced by *AARS2* mutations

Report	Sex	Race	Family history	Age at onset^a^	Age at death	Initial neuropsychiatric symptoms	Cognitive decline	Psychiatric symptoms	Pyramidal symptoms	Extrapyramidal sign	Cerebellar symptoms	Dystonia/Epilepsy	Ovarian failure	Mutation sites
Lynch et al. (2016)	F	NA	NA	early 40s	NA	calculation, anxiety	+	+	+	NA	+	NA	+	c. 1041−1G>A (Exon 6^b^)/c. 595C>T (p. R199C)
	M	Turkey	+	late 30s	NA	neuropsychiatric and behavioral changes	+	+	NA	+	NA	NA	M	c. 1188G>A (Exon 8^b^)/c. 1709delG (p. G570Afs*21)
	M	Turkey	+	Mid‐20s	1 year^c^	neurodegenerative syndrome	NA	−	+	NA	NA	NA	M	c. 1188G>A (Exon 8^b^)/c. 1709delG (p. G570Afs*21)
	M	South Asian	−	middle adolescence	late adolescence	psychiatric symptoms	NA	+	+	+	+	NA	M	c. 892_894del (p. 298_298delQ)/c. 2234_2235del (p. S745Cfs*60)
	M	Turkey	−	Mid‐40s	1 year^c^	dystonia, dysarthria	+	+	+	+	NA	Dystonia	M	c. 595C>T (p. R199C)/c. 595C>T (p. R199C)
Szpisjak et al. (2017)	M	Caucasian	−	18	NA	behavioral changes	+	+	+	+	NA	‐	M	c. 578T>G (p. L193*)/c. 595C>T (p. R199C)
Dallabona et al. (2014)	F	NA	NA	2	NA	developmental delay	+	+	NA	+	+	NA	+	c. 149T>G (p. F50C)/c. 1561C>T (p. R521*)
	M	NA	NA	infancy	NA	nystagmus	+	NA	+	+	+	Dystonia	M	c. 2893G>A (p. G965R)/c. 1213G>A (p. E405K)
	F	NA	NA	33	NA	depression, cognitive deterioration	+	+	NA	+	NA	Epilepsy	+	[c. 1609C>T (p. Q537*)+c. 2350del (p. E784Sfs*9)]/ [c. 595C>T (p. R199C)+c. 2188G>A (p. V730M)]
	F	NA	NA	24	28	tremor	+	+	+	+	+	Dystonia	+	c. 230C>T (p. A77V)/[c. 595C>T (p. R199C)+c. 2188G>A (p. V730M)]
	F	NA	NA	40	46	depression, cognitive deterioration	+	+	−	−	−	‐	+	[c. 595C>T (p. R199C)+c. 2188G>A (p. V730M)]/c. 390_392del (p. F131del)
	F	NA	NA	22	NA	gait problems	−	+	+	NA	+	NA	+	[c. 595C>T (p. R199C)+c. 2188G>A (p. V730M)]/c. 2611dup (p. T871Nfs*21)
Hamatani et al. (2016)	F	Japanese	−	30	NA	cognitive decline, abnormal behaviors	+	+	+	+	+	NA	+	c. 1145C>A (p. T382K)/c. 2255 + 1G>A (?)
Lee et al. (2017)	F	Korean	+	35	NA	cognitive impairment	+	NA	+	+	+	NA	+	c. 963C>A (p. Y321*)/c. 452T>C (p. M151T)
	M	Korean	+	35	NA	mistakes in machine manipulation	+	NA	+	NA	+	Dystonia	M	c. 963C>A (p. Y321*)/c. 452T>C (p. M151T)
This study	M	Chinese	−	40	−	difficult to walk	+	+	+	−	+	‐	M	c. 452T>C (p. M151T)/c. 452T>C (p. M151T)

Abbreviations: F, female; M, male; NA, not available.

a Onset of neuropsychiatric symptoms.

b Exclusion of the indicated exon.

c Died within 1 year of onset of the disease.

Clinical manifestations of the 16 leukodystrophy patients carrying *AARS2* mutations include cognitive decline, psychiatric symptoms, pyramidal symptoms, extrapyramidal signs, cerebellar symptoms, dystonia, and epilepsy (Table 1). These mutations are missense, nonsense, or frameshift by nature. Two of them are homozygous, including a case from Turkey, c. 595C>T (p. R199C) (Lynch et al., 2016), and the c. 452T>C (p. M151T) being reported herein. The probability for this case to occur is rather small given that his biological parents are non‐consanguineous. The mutation was previously reported in a pair of Korean brother‐sister twin, despite having compound heterozygous mutations additionally bearing a nonsense mutation site, c. 963C>A (p. Y321*) (Lee et al., 2017). Due to the small number of reported cases, it is difficult to correlate these mutations with specific clinical phenotypes. Interestingly, although the male patients are absent from any endocrine diseases and hypogonadism, all of the females are diagnosed with ovarian failure, suggesting that analysis of the *AARS2* gene should be prioritized when this sign is displayed in female patients.

## 4 CONCLUSION

In summary, we herein report a Chinese male patient of adult‐onset leukodystrophy carrying a c. 452T>C (p. M151T) homozygous mutation in *AARS2*. It is autosomal recessive based on genotypic analyses of his family members. Our data provide further evidence that mutations of *AARS2* are implicated in adult‐onset leukodystrophy.

## CONFLICT OF INTERESTS

The authors declare that there is no potential conflict of interest.

## AUTHORS' CONTRIBUTIONS

J‐YW, S‐FC, and XZ examined the patient, acquired, and analyzed all clinical data, and interviewed his relatives. J‐YW, H‐QZ, and M‐YW collected and analyzed blood samples and interpreted the genetic data. J‐HZ and J‐YW reviewed literatures and drafted the manuscript. XZ and J‐HZ supervised the study. All authors read, revised and approved the final version of the manuscript.

## ETHICS APPROVAL AND CONSENT TO PARTICIPATE

The study was approved by the Ethics Committee of the Second Affiliated Hospital and Yuying Children's Hospital, Wenzhou Medical University. Written informed consents were obtained from the patient's next‐of‐kin older brother on behalf of the patient and all of the other subjects to participate in the study.

## CONSENT FOR PUBLICATION

The Mini‐Mental State Examination score of the patient was 12/30, indicating moderate‐to‐severe cognitive impairment. As such, written informed consents were obtained from the older brother on behalf of the patient, as well as all of the other participants for publication of this report.

## Data Availability

Data sharing is not applicable to this article as no other data were created or analyzed in this study.
